# Cost-effectiveness of cardiac resynchronization therapy plus an implantable cardioverter-defibrillator in patients with heart failure: a systematic review

**DOI:** 10.1186/s12962-021-00285-5

**Published:** 2021-05-21

**Authors:** Abedin Teimourizad, Aziz Rezapour, Saeed Sadeghian, Masih Tajdini

**Affiliations:** 1 Department of Health Economics, School of Health Management and Information Sciences, Iran University of Medical Sciences, Tehran, Iran; 2 Health Management and Economics Research Center, School of Health Management and Information Sciences, Iran University of Medical Sciences, Tehran, Iran; 3 Tehran Heart Center, Tehran University of Medical Sciences, Tehran, Iran

**Keywords:** Heart failure, Cost-effectiveness, Implantable cardiac devices, Systematic review

## Abstract

**Introduction:**

Heart failure (HF) is an unusual heart function that causes reduction in cardiac or pulmonary output. Cardiac resynchronization therapy (CRT) is a mechanical device that helps to recover ventricular dysfunction by pacing the ventricles. This study planned to systematically review cost-effectiveness of CRT combined with an implantable cardioverter-defibrillator (ICD) versus ICD in patients with HF.

**Methods:**

We used five databases (NHS Economic Evaluation Database, Cochrane Library, Medline, PubMed, and Scopus) to systematically reviewed studies published in the English language on the cost-effectiveness of CRT with defibrillator (CRT-D) Vs. ICD in patients with HF over 2000 to 2020. Consolidated Health Economic Evaluation Reporting Standards (CHEERS) checklist was applied to assess the quality of the selected studies.

**Results:**

Five studies reporting the cost-effectiveness of CRT-D vs ICD were finally identified. The results revealed that time horizon, direct medical costs, type of model, discount rate, and sensitivity analysis obviously mentioned in almost all studies. All studies used quality-adjusted life years (QALYs) as an effectiveness measurement. The highest and the lowest Incremental cost-effectiveness ratio (ICER) were reported in the USA ($138,649per QALY) and the UK ($41,787per QALY), respectively.

**Conclusion:**

Result of the study showed that CRT-D compared to ICD alone was the most cost-effective treatment in patients with HF.

## Introduction

Heart failure (HF) is one of the most common and deadliest disease that consumes the highest rate of health expenditure in the world [1, 2]. It is a set of clinical syndromes in which abnormal cardiac function leads to a decrease in cardiac or pulmonary output or persistent chest pain [3]. Each year, approximately 3.5 million people suffer from HF worldwide, and if left untreated, after one year, death rate rises to about 40 percent [4,7]. According to the European Society of Cardiology (ESC), costs of HF are very high, reaching up to 2% of the total health costs in the developed countries [8].

It is predicted that the total HF cost will increase to $69.7 billion in 2030 worldwide [9].

According to clinical guidelines, there are several procedures to improve HF patient's conditions. Cardiac pacemakers are one of the mechanical devices used to recover heart function [10]. Pacemakers are made of a generator with a small battery which is connected to one or more electrical leads. As soon as the normal heart rate decreases, these pacemakers transfer electrical waves from the generator to the ventricles or atria and cause a series of atrial and ventricular contractions [11]. In HF patients, based on some clinical factors such as, left ventricular ejection fraction (LVEF), ischemic etiology status, QRS duration and New York Heart Association (NYHA) class, Cardiac resynchronization therapy (CRT) helps to recover ventricular dysfunction through pacing the right and left ventricles concurrently [12,15]. Existing clinical guidelines recommend CRT in patients with moderate-to-severe heart failure (NYHA class IIIIV), LVEF (35%) and delayed intraventricular conduction evidenced by a wide QRS complex. Also, the most important clinically question about CRT is whether adding CRT capability to implantable cardioverter-defibrillator (ICD) is marginally cost-effective. The National Institute for Health and Care Excellence (NICE) recommends CRT combined with an implantable cardioverter-defibrillator (CRT-D) to improve outcomes in HF patients with NYHA class II- IV with low ejection fraction (35%) and wide QRS [16, 17].

Due to the high economic burden of heart disease as well as the limited financial resources, not only does it impact individual patient cost, but also the health care system. According to the Dhvani Shah et al. (2020), CRT, CRT-D and ICD devices related cost in US dollars, is $ 17,982, $ 36,153 and $ 23,317 respectively [26]. Several studies have done cost-effectiveness of CRT in HF. But to date, no study systemically reviewed quality appraisal of cost-effectiveness of CRT combined with ICD. So this study developed to systematically review cost-effectiveness of CRT combined with ICD versus ICD alone in patients with HF.

## Methods

### Literature search

This systematic review was conducted to assess articles on the cost-effectiveness of CRT combined with an ICD versus ICD alone in patients with HF between January 2000 and July 2020. A literature search was performed through five reliable databases including NHS Economic Evaluation Database, Cochrane Library, Medline, PubMed, and Scopus. Keywords for searching and identifying relevant studies were: cost-effectiveness or cost-utility or cost-benefit or economic evaluation and heart failure or cardiac failure or myocardial failure or heart decompensation and implantable cardioverter defibrillator or cardiac resynchronization therapy.

### Inclusion and exclusion criteria

Inclusion criteria for studies were: original researches which performed a full economic evaluation, contained cost-effectiveness analysis, cost-utility analysis or cost-benefit analysis, articles which expressed quality-adjusted life years (QALYs), life-years gained or prehospitalization as their outcomes measures. Also studies that evaluated CRT combined with an ICD versus ICD alone in patients with HF, and articles published in the English language during 2000 to 2020.

Also, the exclusion criteria were: studies with a partial economic evaluation (such as those evaluating effectiveness, evaluating costs, and assessing the quality of life, review and meta-analyses articles, studies published as abstracts only, case reports, conferences papers, and low-quality studies according to the Consolidated Health Economic Evaluation Reporting Standards (CHEERS) checklist.

### Quality assessment of the methodology of the studies

The CHEERS statement was used to assess the reporting quality of studies. This checklist consists of 24 recommendations that evaluate the quality of methodology of health care economic assessment studies in the following items: Heading and summary, content and aims, study population and subcategories, place of study, study perspective, comparators, time horizon, discount amount, select of health outcomes, assessment of effectiveness, measurement and estimate of preference-based outcomes estimating resources and costs, currency, type of model, assumptions, analytical methods, study parameters, incremental costs and effects, characterizing uncertainty, characterizing heterogeneity, discussion, source of funding, and disclosures [18]. After searching the studies, the selected articles were assessed by two researchers in terms of the quality of methodology using the CHEERS checklist. Disagreements among two primary reviewers were solved by the third researcher. Finally, according to the CHEERS checklist, the quality score of each study was reported as a percentage (%) out of a total of 24 items.

### Data analysis

Designed data collection forms were used to extract and summarize the required information from the selected studies. Endnote version X7.7 software was used to organize the studies, read the titles, abstracts, and identify duplicates articles. Final selected studies in this review reported a wide range of incremental cost-effectiveness ratio (ICER). In order to compare different ICER, all of them were inflated by 2020 at an annual rate of 3% [19].

## Results

### Search results

**Fig. 1:**
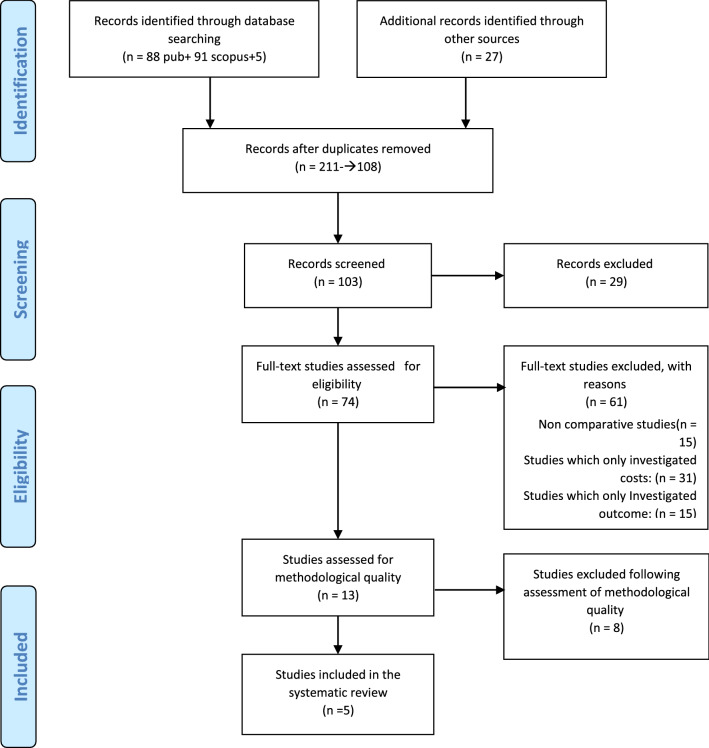
Search results and study selection and inclusion process

A total of 211 studies were identified; duplicated studies were deleted. Then 29 studies were excluded based on the exclusion criteria. A full-text review was conducted for remained articles and 61 of them were excluded. Ultimately, we selected and evaluated the results of five studies with full economic evaluation (cost-effectiveness) of CRT combined with an ICD versus ICD in patients with HF [20,24]. The results of the systematic review are shown in Fig. 1.

### Study characteristics

Characteristics of five selected articles are summarized according to the designed data collection form as follows: first author of the study, country setting, published year, study population, comparator, effectiveness measure, time horizon, type of model used for data analysis, perspective of the study, type of cost used in analysis, type of sensitivity analysis, the discount rate for costs and effectiveness, and the incremental cost-effectiveness ratio (ICER) (Table 1).

**Table 1:** Cost-effectiveness study characteristics

No.	Author	Country and year	Study model	Population	Comparators	Effectiveness measure	Time horizon	Study perspective	Included cost	Sensitivity analyses	Discount rate for cost and effectiveness	ICER (standardized value)
1	Dhvani Shah et al. [24]	USA, 2020	Cohort survival model	N = 12,638 Randomized clinical data real world evidence ( RWE), 4 NYHA classs, 3 QRS categories (< 120 ms, 120 ms/< 150 ms, 150 ms), 4 LVEF categories ( 20, > 20 & 25, > 25 and 30, > 30), 2 Etiology (ischemic/non-ischemic)	CRT-D vs ICD	Life years (LY), QALY	Life time	US payer	Direct medical cost	Deterministic, probabilistic	3%	The incremental cost-effectiveness ratio of CRT-ICD compared to ICD was $100,000/QALY ($100,000/QALY)
2	Mealing et al. [22]	UK, 2016	Decision	N=12,638 A typical UK HFrEF, pateints aged 66 years or older, all NYHA classes and LVEF 35%, 1 etiology (ischemic)	ICD vs CRT-D, CRT-P	QALY	Life time	NHS	Direct medical Cost	Deterministic	3.5%	At a threshold of 30 000 per QALY gained, CRT-D is cost-effective ($41,787/QALY)
3	Woo et al. [20]	USA, 2015	Markov decision	N/A Patients aged 65 years or older, NYHA classes I or II, QRS of 12 msec or more, LVEF of 30% or less, 2 etiology (ischemic/non-ischemic)	CRT-D vs ICD	Life years (LY), QALY	Life time	Societal	Direct medical cost	Deterministic	3%	ICER increased to $119,600 per QALY ($138,649/QALY)
4	Bertoldi et al. [23]	Brazil, 2013	Markov	N=316 Hypothetical cohort of heart failure, NYHA class II, III or IV, prolonged QRS and LVEF 35%, etiology(NA)	CRT-D vs ICD, CRT-P	QALY	20 years	Brazilian public health system	Direct medical cost	Deterministic, probabilistic	5%	For CRT combined with an implantable cardioverterdefibrillator (ICD), ICER was Into $ 36,940/QALY over ICD alone ($45,431/QALY)
5	Noys et al. [21]	USA, 2013	No model	N=1271 (MADIT-CRT) trial, NYHA classes I or II, QRS of 130 msec or more and LVEF of 30% or less,2 etiology (ischemic/non-ischemic)	CRT-D vs ICD	Life years(LY), QALY	4 year follow-up	Third-party payer	Direct medical cost	Deterministic, probabilistic	3%	The incremental cost-effectiveness ratio of CRT-ICD compared to ICD was $58,330/quality adjusted life years(QALY) ($71,738/QALY)

Final five studies in this review have different cost and effectiveness analysis methods, different viewpoints, and different population size in cost-effectiveness evaluation. Also, history and follow-up duration time of patients are dissimilar. Moreover, the value of health spending and the opportunity cost are different between countries. Finally, only English language studies were used in the study inclusion, which means that articles from other languages might have been omitted.

Result of our study revealed that selected studies were conducted in the United States, the United Kingdom, and Brazil. Two studies used the Markov model [20, 23], one study used the decision tree model [22], and in one study, type of model was not mentioned [21]. However, in one study type of model was the Cohort survival model [24]. Discount rate for cost and effectiveness, study perspective, and time horizon of the study were included in all studies. In four studies, discount rate was 3%-3.5% [20,22, 24] while in one study it was 5% [23]. Also, all studies used QALY as effectiveness measurement and in three studies life-years gained added to QALY [20, 21, 24]. Among the final five studies, three of them concurrently conducted deterministic and probabilistic sensitivity analyses [21, 23, 24]. But in two studies deterministic sensitivity analyses were used [21, 23]. Usually, the sensitivity analysis method is used to measure the effect of uncertainty on results and also the generalizability of findings [25,29]. Governmental perspective was used in four studies [21,24] but in one study it was societal [20].

Results from the current study also revealed that all studies applied direct medical costs in their analysis but direct non-medical costs and indirect costs did not contain in these studies.

As a final point, after standardizing value of ICER in selected studies, the highest and the lowest Incremental cost-effectiveness ratio (ICER) were reported in the USA ($138,649per QALY) and the UK ($41,787per QALY), respectively [20, 22].(Table 1).

Also, result of the CHEERS checklist stated that Mealing et al. had the highest satisfied percent (100%) [22] and Woo et al. had the lowest one (88%) [20]. Moreover, Bertoldi et al., Noys et al., and Dhvani Shah et al. had 96, 92, and 83 satisfied percent respectively [21, 23, 24]. (Table 2).

**Table 2:** Results of analysis against CHEERS statement

Author	CHEERS items satisfied	Relevant CHEERS items	Percent satisfied
Dhvani Shah et al. (2020)	22	24	92%
Mealing et al. (2016)	24	24	100%
Woo et al. (2015)	21	24	88%
Bertoldi et al. (2013)	23	24	96%
Noys et al. (2013)	22	24	92%

We also stated other characteristics of the selected studies including: subsections on costs, resource use, methods for HRQOL, methods for treatment effect, summary of health states, life years gained, time in each health state, number of events and Study Calculation Method in Table 3. For example, methods for HRQOL in all five selected studies was EQ-5D. Study Calculation Method in Two studies was Regression equations [22, 24] while in three studies it was ICER [20, 21, 23]. The Highest and the lowest life years gain of CRT-D vs ICD were mentioned in Woo et al. (9.8 years) and Noys et al. (3.61 years) respectively [20, 21]. Methods for treatment effect in four studies was metaanalysis [20, 22,24] and in one study it was MADIT-CRT study [21] (Table 3).

**Table 3:** Cost-effectiveness study- more practical characteristics

No.	Author	Subsections on costs	Methods for HRQoL	Summary of health states	Time in each health state	Number of events	Resource use	Methods for treatment effect	Life years gained	Study calculation method
1	Dhvani Shah et al. [24]	HF-related hospitalizations and other resource use	EQ-5D	NA	Monthly	4 (HF hospitalization, death, health-related quality of life and device-specific treatment effects)	baseline hospitalization risk and Medicare-specific costs	Network meta-analysis	7.97 yrs CRT-D VS 7.47 yrs ICD	Regression equations
2	Mealing et al. [22]	Hospitalizations costs and cross-manufacturer average selling prices for both intervention and leads	EQ-5D	NA	Yearly	4 (mortality, hospitalization , health-related quality of life, and device-related effects)	National databases for costs and clinical literature and expert opinion	Network meta-analyses	NA	Individual patient based regression
3	Woo et al. [20]	HF-related hospitalizations and costs related to device implantation, extraction, reimplantation	EQ-5D	Stable, hospitalization, lead failure, lead infection, death	Monthly	4 (Successful with no complications, Successful with complications, Not successful, Procedural death)	Clinical trials, clinical registries, claims data from centers for medicare and medicaid services, and centers for disease control and prevention life tables	Meta-analysis of trials	9.8 yrs CRT-D VS 8.8 yrs ICD	ICER
4	Bertoldi et al. [23]	costs of consultations, diagnostic tests, procedures, hospital admissions, device implantation and complications	EQ-5D	Stable, lead failure, hospitalization, lead infection, death	Monthly	3 (failure, success, death)	Brazilian Ministry of Health (public healthcare system)	Meta-analysis of trials	7.54 yrs CRT-D VS 6.9 yrs ICD	ICER
5	Noys et al. [21]	Hospitalization for implantation, inpatient and outpatient costs, emergency room	EQ-5D	NA	Every 6 month	2 (HF events, death)	National medicare reimbursement rates	MADIT-CRT study	3.61 yrs CRT-D VS 3.54 yrs ICD	ICER

We noticed that Dhvani Shah et al. has used underlying economic model of Mealing et al. in different perspective, effectiveness measure and discount rate.

## Discussion

This systematic review evaluated the results of five relevant articles which concluded CRT plus ICD in HF patients might be more cost-effective than ICD alone. It means that if policy makers want to use results of our study, they should consider to the clinical heterogeneity in different patient groups. Cost-effectiveness analysis is a method to measure resource consumption related to a health intervention. In these studies, a specific health care intervention is compared with available alternatives in terms of effectiveness and costs. This helps to better resource allocation in the health care system.

There are two types of CRT devices: biventricular pacemaker CRT, and biventricular pacemaker with defibrillator CRT-D. Also, ICD is a cardiac defibrillator [30]. In patient with HF, most of the candidates for CRT devices also have an indication for an ICD. So these patients most commonly receive a CRT-D device compared to a CRT [31]. Results of some researches showed that CRT-D implantation was highly prevalent (75%) in subgroups that ICD benefit may be reduced (older adults with multiple comorbidities) [32].

The results of our study showed that utility values used in the selected studies were extracted from literature review findings which could not exactly reflect the QALY score. However, ICER Values were reported differently from one study to another. According to Woo et al. (2015), ICER was $119,600 per QALY [20]. Noys et al. (2013) reported it as $58,330 per QALY [21]. ICER was estimated $ 36,940 per QALY by Bertoldi et al. [23]. Dhvani Shah et al. showed that ICER was $100,000 per QALY [24].

In cost-effectiveness studies, ICER may be affected by several factors, such as age and gender of population study, threshold values in different countries, heart diseases epidemiology, costs and effectiveness measurement methods, and fees of intervention and medical equipment in different countries [33].

Moreover, based on the results of the current systematic review, more expensive CRT-D devices, shorter CRT-D battery life, and older age of patients made the cost-effectiveness of CRT-D less advantageous.

Also finding of our study showed that using CRT-D increased effectiveness and reduced costs. Mealing et al. showed that CRT-D reduced monthly hospitalization rate by 30% while ICDs decreased monthly hospitalization rates by 20% [22]. Woo et al. also revealed that CRT-D increased life expectancy (9.8 years versus 8.8 years) and QALYs (8.6 years versus 7.6 years) compared with implantation of an ICD alone [20]. Bertoldi et al. showed that in more flexible healthcare budgets system, CRT-D would be a more attractive option than ICD alone. It means that CRT-D is the preferred strategy in the most liberal budgets (CRT-D is preferred compared to ICD with WTP thresholds above Int$ 35,000). In fact, if the cost of the CRT-D devices was lowered or their battery longevity increased, it would become a more attractive option, with an acceptable ICER considering the World Health Organization WTP threshold [23].

In most studies, major cost drivers were physicians, drugs, diagnostic tests and procedures cost, and also hospitalization costs related to device implantation. Also, results of this systematic review showed that studied population was asymptomatic patients with a left ventricular ejection fraction (LVEF) of 30% or less and QRS duration of 120 ms or more in almost studies. Moreover, results revealed that although early device implantation imposed some cost on patient, survival rate increased in their lifetime.

## Conclusion

CRT-D compared with ICD alone was cost-effective treatment for HF. Also, because the prevalence of HF is high in poor-income countries, it seems more studies should be conducted on the economic evaluation of CRT-D. Because of growing use of cardiac devices especially ICD in low and middle-income countries, the results of our study can be useful for policymakers and HF treatment centers in that countries to apply experiences of developed nations if they want to expand cardiac devices surgeries in the future. Thus, policymakers and clinical specialists in their studies in low and middle-income countries should be noted to different epidemiology and economic circumstance in their setting.

## Data Availability

Not applicable.
